# Roles of N-Methyl-D-Aspartate Receptors (NMDARs) in Epilepsy

**DOI:** 10.3389/fnmol.2021.797253

**Published:** 2022-01-07

**Authors:** Shuang Chen, Da Xu, Liu Fan, Zhi Fang, Xiufeng Wang, Man Li

**Affiliations:** Department of Neurology, Union Hospital, Tongji Medical College, Huazhong University of Science and Technology, Wuhan, China

**Keywords:** *N*-methyl-*D*-aspartate receptor, epilepsy, anti-NMDAR encephalitis, D-serine, glutamate, excitotoxicity, CREB, epigenomics

## Abstract

Epilepsy is one of the most common neurological disorders characterized by recurrent seizures. The mechanism of epilepsy remains unclear and previous studies suggest that N-methyl-D-aspartate receptors (NMDARs) play an important role in abnormal discharges, nerve conduction, neuron injury and inflammation, thereby they may participate in epileptogenesis. NMDARs belong to a family of ionotropic glutamate receptors that play essential roles in excitatory neurotransmission and synaptic plasticity in the mammalian CNS. Despite numerous studies focusing on the role of NMDAR in epilepsy, the relationship appeared to be elusive. In this article, we reviewed the regulation of NMDAR and possible mechanisms of NMDAR in epilepsy and in respect of onset, development, and treatment, trying to provide more evidence for future studies.

## Introduction

Epilepsy is one of the most common neurological disorders characterized by recurrent seizures. Long-term recurrent seizures could lead to cognitive impairment and mental disorders, which severely affect the social interaction level and employment ability of epileptic patients, and result in a decline in the quality of life (Chen et al., 2020a). At present, antiepileptic drugs (AEDs) remain the main therapy of epilepsy, despite no response in about 1/3 patients with epilepsy (Moshé et al., 2015). The mechanism of epilepsy remains unclear but it is generally regarded as a self-facilitated pathological process triggered by brain injury, ultimately resulting in nerve damage, mossin fibrosis, synaptic plasticity, inflammatory response, and ionic pathway dysfunction (Gan et al., 2015). It is widely acknowledged that abnormal excessive synchronous discharge, i.e., the imbalance between excitation and inhibition of neurons, plays an essential role in epileptogenesis.

However, the factors affecting this imbalance were sophisticated, and excitatory amino acids were supposed to participate in this imbalance (Bonansco and Fuenzalida, 2016).

In the central nervous system (CNS), N-methyl-D-aspartate receptor (NMDAR) is one of the main excitatory receptors on the synapses of neurons including glutamatergic neurons and GABAergic interneurons, which regulate the balance between neuronal excitation and inhibition (Hendry et al., 1987; Hanada, 2020). Meanwhile, NMDARs, the ionotropic glutamate receptors in the brain, are involved in neuroplasticity, excitatory neurotransmission, and neurotoxicity (Fricker et al., 2018; Horak et al., 2021). Related studies have shown that overexcitation of NMDAR leads to neuronal death in neurological diseases such as epilepsy, stroke, Alzheimer’s disease (AD), and Parkinson’s disease (PD; Essiz et al., 2021). In the brain, NMDARs are hetero-tetramers generally composed of two GluN1 subunits and four distinct GluN2 (GluN2A-D) or a mixture of GluN2 with two different GluN3 (GluN3A and 3B) subunits. The GluN1 subunit is required for NMDAR activation and binds to the necessary co-agonists through the amino-terminal domain of the extracellular region. GluN2 subunits are able to bind glutamate specifically and these subunits are different from each other by providing different functional properties of NMDAR. In the NMDAR, *GRIN1* codes for GluN1 subunit, *GRIN2* codes for GluN2 subunit, and *GRIN3* codes for GluN3 subunit (Beesley et al., 2020). Meanwhile, the triheteromer (GluN1/GluN2A/GluN2B) is the main subtype of NMDAR and is widely expressed in the cortex and hippocampus (Luo et al., 1997; Tovar et al., 2013).

Previous studies have shown that glutamate levels increase in the extracellular fluid during seizures in temporal lobe epilepsy (TLE) and glutamate can directly activate NMDAR and induce neuroexcitatory toxicity (Albrecht and Zielińska, 2017). Meanwhile, it has been reported that NMDA, AMPA, and kainite (KA) can induce seizures in animal models, and glutamate receptor antagonists inhibit seizures in animals (Celli and Fornai, 2020). In the PTZ-induced status epilepticus (SE), GluN1, GluN2A, and GluN2B subunits are increased and synaptic plasticity impairs in the hippocampus of rats. Meanwhile, the increase in the GluN2B subunit may result in the decrease of long-term potentiation (LTP; Postnikova et al., 2017). Related studies have shown that *GRIN1*, *GRIN2A*, and *GRIN2B* mutations can lead to epilepsy. In all mutations, *GRIN2A* variants are associated with neurological diseases including developmental and epileptic encephalopathy, which may be manifested as seizures, mild speech and language delay, and cognitive impairment (Lemke et al., 2013, 2014; Fry et al., 2018). In addition, anti-NMDAR encephalitis, a major type of autoimmune encephalitis (AE), has been reported to be an entity of epilepsy (Leypoldt et al., 2015). In addition to neurotoxicity, NMDARs can also participate in neuroprotection by activating cAMP response element-binding protein (CREB) signals in epilepsy (Wang et al., 2020b).

**Figure 1:**
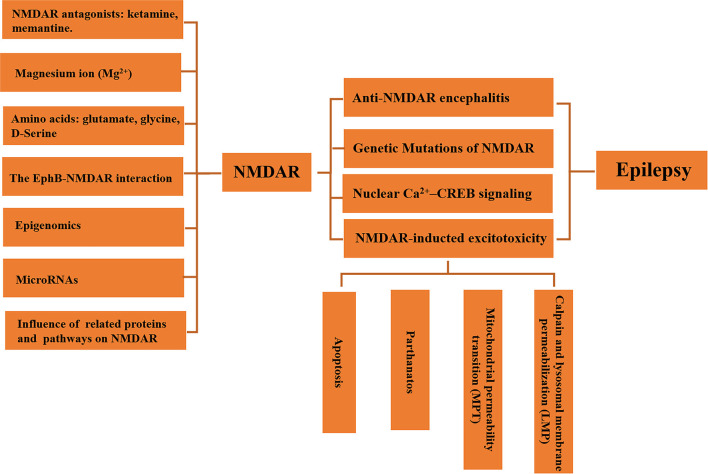
Regulation of neuroexcitatory receptor N-Methyl-D-Aspartate Receptor (NMDAR) in epilepsy.

Owing to its role in brain functional plasticity and neuroexcitatory, the regulation of NMDAR in epilepsy has attracted extensive attention. As noted above, NMDARs have been shown to involve in seizures, but functions and mechanisms of NMDARs in epilepsy appear to be elusive. In clinical study, understanding the function of NMDAR is of great significance for the treatment of epilepsy and AEDs selection. This article reviews the regulatory mechanism of NMDAR and the progress of NMDAR in the occurrence, development, and treatment of epilepsy from various points of view (Figure 1).

## NMDAR and Epilepsy

### Anti-NMDAR Encephalitis

Anti-NMDAR encephalitis is a major type of AE in which the over-production of NMDAR autoantibodies results in profound neurotransmitter dysregulation, causing seizures and other symptoms such as dysautonomia, orofacial dyskinesia, psychosis, mental status changes, hallucinations, and headaches (Tong et al., 2020). Related studies have shown that approximately 75% of patients with anti-NMDAR encephalitis develop seizures, and refractory status epilepticus (RSE) can lead to neuronal death (Geis et al., 2019). However, the mechanism of anti-NMDAR encephalitis leading to seizures is not fully understood.

NMDARs have been reported to play a role in synaptic homeostasis. Related studies have found that NMDAR autoantibodies could increase extracellular glutamate levels in the brain (Manto et al., 2010). Some findings also have confirmed that NMDAR autoantibodies can result in the internalization of surface NMDARs and the decreasing of receptor density in the patients with anti-NMDAR encephalitis (Hughes et al., 2010; Takahashi et al., 2020). In the patients, NMDAR autoantibodies bind and cross-link to a specific region of NMDARs GluN1, then internalize NMDARs (Gleichman et al., 2012). NMDAR-EphB2 interaction plays a key role in the NMDAR autoantibody-mediated NMDAR internalization. When autoantibodies bind to endogenous NMDARs, the interaction between NMDAR and EphB2 is disrupted, thereby leading to NMDAR internalization and dysfunction, and the reduction of NMDAR-mediated synaptic currents (Hughes et al., 2010; Mikasova et al., 2012). After activation of EphB2, the extracellular domain of EphB2 interacts directly with the GluN1 subunit, thereby stabilizing NMDARs in the synapse (Dalva et al., 2000; Washburn et al., 2020). Meanwhile, a related report has indicated that NMDAR autoantibody interferes with the interaction between NMDARs and EphB2 in cultured hippocampal neurons (Mikasova et al., 2012). In addition, cerebrospinal fluid (CSF) of the patient with anti-NMDAR encephalitis can also result in the reduction of both GluN2A and GluN2B on the synaptic surface and prevent a chemically induced LTP of glutamate synapses (Mikasova et al., 2012). Similar to the anti-NMDAR antibodies-mediated effect, the amino terminal domain (ATD) peptide of GluN1 subunit can also actively immunize against NMDARs and induce anti-NMDAR encephalitis in a mouse model (Ding et al., 2021). A study shows that a single injection of anti-NMDAR antibodies from the patient with anti-NMDAR encephalitis into mice does not induce seizures (Wright et al., 2015). However, injection of anti-NMDAR antibodies *in vivo* can increase the number of seizures in the PTZ induced-mice model. Moreover, about 75–93% of mice developed epilepsy after long-term infusion of CSF or purified anti-NMDAR antibodies from patients with anti-NMDAR encephalitis (Wright et al., 2015). It is puzzling that the function reduction of NMDAR is more likely to activate the persistent abnormal discharge of neurons, but a specific inhibition of the NMDARs in the GABAergic interneurons can also explain this phenomenon (Manto et al., 2010). NMDAR autoantibodies reduce the excitability of GABAergic interneurons through the interaction with NMDAR, thereby weakening the inhibitory effect on excitatory transduction of glutamatergic neurons (Geis et al., 2019). The disinhibition of excitatory glutamatergic neurons may also account for seizures in anti-NMDAR encephalitis. In addition, it has been found that ketamine, an NMDAR antagonist, may be useful in super-refractory status epilepticus (SRSE) developed in patients with anti-NMDAR encephalitis (Santoro et al., 2019). Despite the profound effect on NMDARs, there is no evidence that NMDAR autoantibodies can alter the localization and expression of other glutamate receptors such as AMPARs, the synaptic protein PSD-95, as well as the number of synapses, or affect the survival of nerve cells *in vitro* or *in vivo* models (Huang and Xiong, 2021). In a word, both the activation of NMDARs in glutamatergic neurons and the inhibition of NMDARs in GABAergic interneurons may participate in epileptogenesis and more researches are urgently needed.

### NMDAR in Epilepsy

In recent years, NMDAR subunit-encoding genes have been confirmed to be involved in epilepsy and the genetic mutations in NMDARs may cause epilepsy in humans, suggesting that NMDAR is closely related to epilepsy (Xu and Luo, 2018). Besides, impairment of NMDAR signals as a result of genetic or environmental insults leads to a variety of neurodevelopmental disorders, including epilepsy, schizophrenia, intellectual disability, or autism (Mielnik et al., 2021). Meanwhile, NMDAR-mediated excitotoxicity was supposed to participate in neuronal death induced by high levels of glutamate and aspartate in neurological diseases such as epilepsy, stroke, AD, and PD (Fricker et al., 2018; Essiz et al., 2021). This article will review genetic mutations of NMDAR, signaling pathways of NMDAR-mediated excitotoxicity, and NMDAR-dependent neuroprotection in epilepsy.

#### Genetic Mutations of NMDAR in Epilepsy

GluN1 subunit, the essential subunit of functional NMDAR is encoded by GRIN1, and *GRIN1* mutations have a significant effect on neuronal activity, causing various types of epilepsy, including SE, focal dyscognitive seizures, myoclonic seizures, febrile seizures, spasms, hypermotor seizures, tonic and atonic seizures, generalized seizures, etc (Wyllie et al., 2013; Fry et al., 2018). Besides, the epileptic phenotype may contribute to the p. Met641Leu *de novo* variant in *GRIN1* gene, and *de novo* *GRIN1* mutations were gradually recognized to be in association with severe early infantile encephalopathy (Pironti et al., 2018). The common characteristics are extensive bilateral polymicrogyria with intractable epilepsy, cortical visual impairment, postnatal microcephaly, and severe developmental delay in patients with *de novo* *GRIN1* mutations (Fry et al., 2018). Extensive bilateral polymicrogyria is associated with severe developmental delay and intractable epilepsy. At present, a variety of polymicrogyria-associated mutations have been found, including p.Asn674Ile, p.Arg794Gln, p.Arg659Trp, p.Asp789Asn, p.Tyr647Cys, p.Asn650Ile, p.Ala653Gly, p.Leu551Pro, p.Ser553Leu, etc (Fry et al., 2018). In *GRIN1* mutations, the mechanisms remained unclear but disrupted gating of the ion channel by p.Gly827Arg mutation and disruption of NMDAR ligand binding by p.Ser688Tyr mutation may be concerned (Zehavi et al., 2017).

Current studies have found that GluN2 subunits may control epileptiform events in the hippocampus (Punnakkal and Dominic, 2018). *GRIN2A*, which encodes the GluN2A subunits, is widely considered to be epileptogenic. The most common types of seizure caused by *GRIN2A* mutations include atypical benign partial epilepsy, Landau–Kleffner syndrome (LKS), and benign epilepsy with centro-temporal spikes (BECT; Hanada, 2020). GluN2 subunits mainly regulate the open/close of the NMDARs. GluN2A-containing receptors have a reversible calcium-dependent inactivation, whereas GluN2B does not (Franchini et al., 2020). Meanwhile, GluN2A subunits can regulate neuronal NMDAR-induced microglia-neuron physical interactions (Eyo et al., 2018). Related studies have shown that voltage-independent GluN2A-related NMDAR-Ca^2+^ signaling is related to audiogenic seizures, attentional and cognitive deficits in mice (Bertocchi et al., 2021). A rare variant of *GRIN2A* associated with epilepsy disrupts CaMKIIα phosphorylation of GluN2A and NMDAR trafficking, which demonstrates a role of GluN2A for CaMKIIα phosphorylation in receptor targeting and suggests that the defects of NMDAR trafficking are related to epilepsy (Mota Vieira et al., 2020). There were defects of GRIN2A related to epileptiform discharges and transient microstructural brain abnormalities in mice with epilepsy (Salmi et al., 2018). The mutant GluN2A (p.Met817Val)-containing receptors decreased sensitivity to endogenous negative inhibitors (Mg, zinc), prolonged the time of synaptic response, increased the time of single-channel mean open, and the probability of channel open. These acquired *GRIN2A* mutations lead to overactivation of NMDAR and increase neuronal excitability, which may be related to epileptogenesis observed in patients (Chen et al., 2017). A *de novo* *GRIN2A* missense mutation (p.Asp731Asn) in a child with focal epilepsy and acquired epileptic aphasia was reported. However, this mutant reduced NMDAR activation, suggesting that NMDAR hypofunction may also be related to epilepsy pathogenesis (Gao et al., 2017).

*GRIN2B* mutation is a rare cause of severe epileptic encephalopathy (Sharawat et al., 2019). A related study demonstrated that *GRIN2B, BDNF*, and *IL-1β* gene significantly were upregulated and *GRIN2B* was positively correlated with the expressions of *BDNF* and *IL-1β* gene in people with epilepsy (Zhand et al., 2018). Some *GRIN2B* mutations (p.Val618Gly and p.Asn615Ile) were found in patients with early-onset epilepsy and epileptic encephalopathy (Lemke et al., 2014; Smigiel et al., 2016). Those GluN2B heteromers showed a significant loss of ion-channel block by extracellular Mg^2+^ and a significant increase of Ca^2+^ permeability (Lemke et al., 2014). Meanwhile, blocking GluN2B-containing NMDARs can reduce short-term brain injury caused by early-life SE (Loss et al., 2019).

In addition, *GRIN2C* expression is limited to astrocytes whereas *GRIN2D* are expressed at high levels in GABAergic interneurons in the hippocampus (Shelkar et al., 2019; Dubois and Liu, 2021). These specific distributions are likely to be highly relevant to mechanisms of epilepsy and dysregulation of glutamatergic signaling. Some *GRIN2D* variants (p. Thr674Lys, p.Met681Ile, p.Ser694Arg, p, Asp449Asn, p.Val667Ile, p.Ser573Phe, p.Leu670Phe, p.Ala675Thr, p.Ala678Asp, p.Ser1271Leu, and p.Arg1313Trp) have been found in developmental and epileptic encephalopathy (Tsuchida et al., 2018; Jiao et al., 2021). In a novel *GRIN2D* variant with epileptic encephalopathy, *GRIN2D* mutation-related epilepsy is found to be refractory to conventional AEDs (Camp and Yuan, 2020; Jiao et al., 2021). However, *GRIN2D* dominant mutations can cause severe epileptic encephalopathy, which can be treated with NMDAR channel blockers (Li et al., 2016). In epilepsy, we need to further understand the unique characteristics of *GRIN2D* mutations in neurological function and pathology, which is conducive to the treatment of refractory epilepsy.

#### NMDAR Mediates Excitotoxicity in Epilepsy

**Figure 2:**
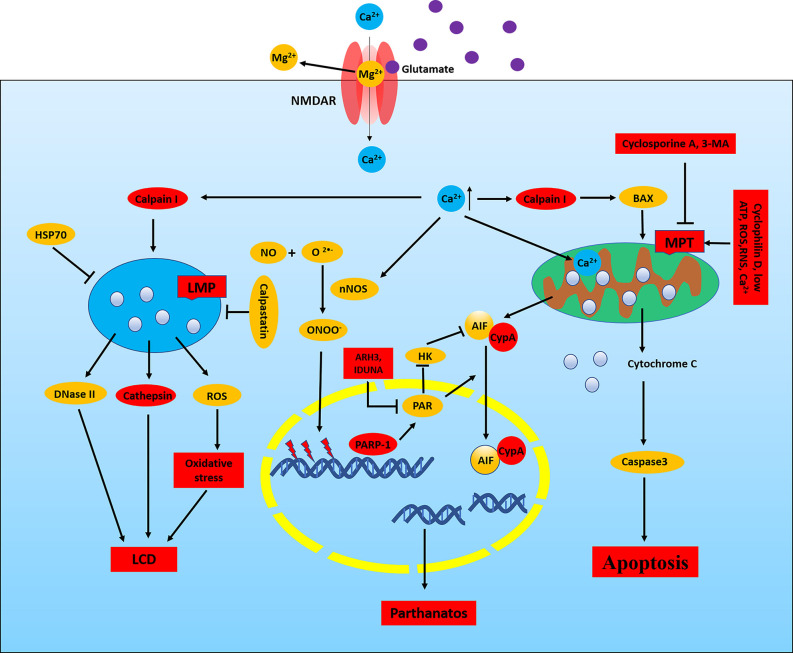
NMDAR-mediated excitotoxicity in epilepsy. In neurons, the NMDAR channel is blocked by Mg^2+^ at neuronal resting membrane potential, and Mg^2+^ is removed when the membrane is depolarized. Activated NMDAR leads to calcium loading which will cause the activation of nNOS, calpain I, and mitochondrial permeability transformation (MPT) pore and eventually lead to neuronal death. Calpain I can cleave Bid and Bax, leading to the release of apoptosis-inducing factor (AIF) and cytochrome C from the mitochondria. Meanwhile, cytochrome C can induce the activation of caspase, and calpain I can also directly cleave and activate caspases, thus resulting in apoptosis. In addition, AIF is cleaved by calpain I to a tAIF, which translocates to the nucleus and induces DNA cleavage, thereby leading to apoptosis and parthanatos. Activation of calpain can cause lysosomal membrane permeability (LMP), which releases the toxic cathepsin, DNase II, and ROS, thereby resulting in LCD. Meanwhile, HSP70 and calpastatin can resist LMP. Increased Ca^2+^, ROS, RNS, and low ATP in mitochondrial matrix results in MPT which depends on the opening of mPTP. Cyclosporine A and 3-MA can block MPT. Ca^2+^ directly activates nNOS, which can catalyze NO and O_2_^−^ to form ONOO^−^. ONOO^−^ damages DNA, thereby activating PARP1, resulting in parthanatos. PARP1 is involved in chromosomal stability, DNA repair, and inflammatory responses. PAR, the product of PARP1 activity, induces nuclear translocation of AIF and inhibits HK. Nuclear translocation of AIF requires the involvement of CypA, which binds to AIF and forms CypA-AIF complex after the release from mitochondria, thereby participating in DNA degradation and leading to parthanatos. ARH3 reduces PAR levels in the nucleus and cytoplasm and IDUNA reduces the release of AIF by binding to the PAR polymers and prevents PARP1-induced cell death. LCD, lysosomal cell death; HSP70, heat shock protein 70; HK, hexokinase.

In the CNS, high levels of glutamate induce neuronal death by NMDAR-mediated excitotoxicity (Olney, 1971). Glutamate-induced excitotoxicity is mainly attributed to apoptosis, autophagy, parthanatos, phagocytosis, ferroptosis, apoptosis- inducing factor (AIF), calpain I, mitochondrial permeability transformation (MPT), lysosomal membrane permeability (LMP), and RNS and ROS production (Figure 2; Fricker et al., 2018). Activated NMDARs lead to neuronal depolarization and calcium (Ca^2+^) loading. The increasing of cytoplasmic Ca^2+^ can cause the activation of nNOS, calpain I, and MPT pore, eventually leading to neuronal death (Figure 2; Fricker et al., 2018). Overexcitation of NMDAR leads to neuronal death in neurological diseases such as epilepsy, stroke, AD, and PD, and blockade of NMDARs can reduce neuronal death in the brain (Essiz et al., 2021).

##### Calpain and Lysosomal Membrane Permeabilization (LMP)

Activated NMDAR leads to Ca^2+^ influx, which activates calpain. Calpain I is involved in the late phase of neuronal death caused by mitochondrial dysfunction. Calpain I, a cysteine protease highly expressed in neurons, is activated by high levels of Ca^2+^ in the cytoplasm and can cleave Bid and Bax, leading to the release of AIF and cytochrome C from the mitochondria (Wang, 2000; D’Orsi et al., 2012). Meanwhile, the release of cytochrome C can induce the activation of caspases, and activated calpain I can also directly cleave and activate caspases, thus resulting in apoptosis (Wang, 2000). However, AIF is cleaved by calpain I to a truncated AIF (tAIF), which translocates to the nucleus and induces DNA cleavage, thereby leading to apoptosis and parthanatos (Fricker et al., 2018). In addition, GluN2A subunit-containing synaptic NMDARs preferentially activates calpain I, which is conducive to neuronal survival by selectively degrading the protein phosphatase PHLPP1α and PHLPP1β (Wang et al., 2013). On the contrary, calpain II is selectively activated by GluN2B subunit-containing extrasynaptic NMDARs and calpain II participates in neuronal death by degrading the protein tyrosine phosphatase STEP (Hoque et al., 2016). Further studies are needed to clarify the role of these two isoforms of calpains in excitotoxic neuronal death.

Activated calpain can also cause LMP, thereby releasing the toxic cathepsin into the cytoplasm and leading to lysosomal cell death (LCD), also known as autolysis in neurodegenerative diseases (Fricker et al., 2018). It has been found that ischemia can induce calpain I to be localized in lysosomes and cause neuronal LMP (Yamashima et al., 1998; Windelborn and Lipton, 2008). Meanwhile, multiple stimuli can also induce LMP, release cathepsin, and induce cell death through a variety of pathways. However, activated NMDAR may also lead to LMP, which is dependent on the activation of calpain (Yan et al., 2016). Activated calpain I can also cleave and inactivate Na^+^/Ca^2+^ exchangers in the plasma membrane of the nerve cells during neuroexcitatory toxicity, thereby leading to calcium overload and necrosis (Bano et al., 2005). Thus, calcium overload and neuronal death can be effectively inhibited by inhibiting calpain. In addition, the absence of calpastatin, a natural calpain inhibitor expressed in neurons, makes neurons more susceptible to excitotoxicity, and its overexpression inhibits neuronal death attributed to excitotoxicity (Descloux et al., 2015). Meanwhile, heat shock protein 70 (HSP70) also stabilizes lysosomes to resist LMP (Aits and Jäättelä, 2013). In conclusion, NMDAR can promote activation of calpain and LMP, while inhibition of calpain and LMP may be an effective method to reduce neuronal death caused by NMDAR-mediated excitotoxicity in epilepsy.

##### Mitochondrial Permeability Transition (MPT)

Activated NMDARs can also result in MPT by Ca^2+^ influx, involved in neuronal death. MPT is characterized by a significant increase in the permeability of the inner mitochondrial membrane with increased Ca^2+^ concentration, which eventually leads to oxidative phosphorylation decoupling, depletion of cell energy, and necrotic cell death. MPT largely depends on the opening of mitochondrial permeability transition pore (mPTP; Fricker et al., 2018). However, increased Ca^2+^ concentration in the mitochondrial matrix is an important factor leading to the opening of mPTP. In addition, it is also closely associated with ROS and RNS, the decreasing of ATP, the decreasing of mitochondrial membrane potential, and intracellular acidification (Fricker et al., 2018). The opening of mPTP leads to the depolarization of the inner mitochondrial membrane, the reduction of ATP production and the increasing of ATP consumption, the rupture of the mitochondrial membrane and the release of cytochrome C and cytochrome G, eventually causing irreversible cellular respiratory arrest and cell death (El-Mir et al., 2008; Fricker et al., 2018). Activated NMDARs can induce MPT by increasing Ca^2+^, ROS, and RNS in neurons, while cyclosporine A can reduce neuronal death by blocking MPT (Schinder et al., 1996). Meanwhile, MPT is also inhibited by 3-methyladenine (3-MA), an inhibitor of autophagosome formation, which can inhibit kinases to regulate neuronal survival and death (Xue et al., 2002). Correlative experimental data showed that cyclosporine A had different protective effects on excitotoxicity induced hippocampal nerve cell death (Santos and Schauwecker, 2003). Thus, blocking the opening of mPTP by cyclosporine A or the gene knockout of cyclophilin D can partially prevent neuronal death caused by excitotoxicity. In addition, studies have shown that high levels of Ca^2+^, ROS, and low levels of ATP in the cytoplasm can promote MPT in the brain of epilepsy, and ketone bodies also mediate antiepileptic effects through MPT (Kim et al., 2015). These studies suggest that MPT may play an important role in the occurrence and treatment of epilepsy.

##### Parthanatos

Ca^2+^ enters the cytoplasm by activating NMDARs and directly activates nNOS which is significantly expressed in the cytoplasm of some GABAergic neurons of the hippocampus and cortex. Activated nNOS can catalyze the reaction of NO with O_2_^−^, thereby producing peroxynitrite (ONOO^−^), which interacts with DNA, lipids, and proteins through a direct oxidative stress response, leading to parthanatos or apoptosis (Figure 2; Conrad et al., 2016; Ivanova V. et al., 2020). Parthanatos is an important form of cell death, characterized by dependence on the overactivation of the nuclear protein PARP1 after DNA damage and ROS production (Virág et al., 2013).

Related studies have shown that glutamate is involved in inducing neuronal injury through the activation of PARP-1 and generation of poly-ADP-ribose (PAR) polymer, thereby participating in parthanatos (Andrabi et al., 2011). In brief, activated NMDARs can promote ONOO^−^ production by activating nNOS, which damages DNA and activates PARP, eventually resulting in parthanatos.

As is well-known, PARP1 and PARP2 are involved in chromosomal stability, DNA repair, and inflammatory responses (Curtin and Szabo, 2013). In PARP-dependent death, activated PARP1 results in production and NAD^+^ depletion. The direct interaction between AIF and PAR promotes the nuclear translocation of AIF, which leads to chromatin degradation (Andrabi et al., 2008; Wang et al., 2011). Meanwhile, PAR is the product of PARP1 activity and also induces nuclear translocation of AIF by inhibiting hexokinase (HK; Wang et al., 2009). Overactivated PARP1 leads to NAD^+^ depletion that further disrupts cellular metabolic processes and promotes cell death (Alano et al., 2010). Nuclear translocation of AIF also requires the involvement of cyclophilin A (CypA), which binds to AIF after the release from mitochondria and forms CypA-AIF complex, thereby participating in DNA degradation under various cellular stress conditions, such as cerebral hypoxia-ischemia and traumatic brain injury (TBI; Zhu et al., 2007; Farina et al., 2017). Related studies have shown that inhibiting the formation of the CypA-AIF complex can reduce glutamate-induced HT22 hippocampal cell death (Doti et al., 2014). The release of AIF in mitochondria may also be associated with calpain, BH3-only protein Bid, and BNIP3 (Fricker et al., 2018). In addition, PAR levels are also regulated by the ADP-ribosyl-acceptor hydrolase 3 (ARH3), which reduces PAR levels in the nucleus and cytoplasm (Mashimo et al., 2013). Meanwhile, the protein IDUNA, also known as E3 ubiquitin protein ligase RNF146, binds to the PAR polymers, thereby reducing the release of AIF and preventing PARP1-induced cell death (Andrabi et al., 2011).

Glutamate acting on NMDAR induces neuronal injury through activation of PARP-1 and generation of PAR polymer (Andrabi et al., 2011). There is currently considerable evidence supporting the role of parthanatos in a variety of neurological disorders including epilepsy, stroke, PD, and TBI, and the inhibition of PARP-1 and PARP-2 can reduce nuclear translocation of AIF and increase neuroprotection (D’Orsi et al., 2016; Xu H. et al., 2019; Dionísio et al., 2021; Koehler et al., 2021). Related studies have found that the formation of the neuronal AIF-CypA complex is considered to be the main target for the recovery of ischemia-stroke injury (Farina et al., 2018). However, in the hippocampal neuronal culture (HNC) model of acute acquired epilepsy, activation of PARP-1 is thought to be a major cause of caspase-independent cell death (Wang S. et al., 2013). Meanwhile, PARP-1-mediated mitochondrial dysfunction promotes neuronal damage in the hippocampus after SE (Lai et al., 2017). In addition, inhibition of PARP-dependent cell death pathways has been shown to prevent seizure-induced neuronal damage (D’Orsi et al., 2016). In epilepsy, activated NMDAR may damage DNA and activate PARP. Thus, blocking PARP-dependent cell death pathways may be a way to mitigate NMDAR-mediated excitotoxicity in epilepsy.

##### Other Signaling Pathways in NMDAR-Mediated Excitotoxicity

In addition to the signaling pathways described above, NMDAR regulates nerve cell death through other pathways. Activated NMDAR can promote the NADPH oxidase (NOX) to produce O_2_^−^ in neurons, leading to neuronal death. A related study has shown that seizures are induced by NMDAR-mediated activation of NOX-induced oxidative stress and can be arrested by NOX inhibition (Malkov et al., 2019). Meanwhile, activated NMDAR increases c-Jun abundance in several neurodegenerative disorders and following ischemia and SE. Phosphorylated c-Jun is transcriptionally active and can induce apoptosis by upregulation of cell death-inducing genes or by downregulating anti-apoptotic genes (Kravchick et al., 2016). In addition, NMDAR overactivation activates NF-κB signaling to promote IL-1β and IL-6 macrophage marker expression. NMDAR silencing and calpain inhibition reduce inflammatory responses (Cheng et al., 2020). It has been reported that glutamate can reduce the insulin-like growth factor-1 (IGF-1) signal through GluN2B-containing NMDAR in cultured cortical neurons, which is considered to be a new mechanism of glutamate-induced neurotoxicity (Zhao et al., 2020). In epilepsy, increased IGF-1 levels after recurrent hippocampal neuronal discharges might promote seizure by IGF-1R-dependent signaling pathways (Jiang et al., 2015). However, the previous role of the NMDAR-IGF-1 signal is unappreciated in the development of seizure activity.

#### NMDAR-Dependent Neuroprotection in Epilepsy

Ca^2+^ influx could stimulate and induce cell death, but it is also in association with NMDAR-dependent neuroprotection (Wang et al., 2020b). Ca^2+^ entering the cytoplasm *via* synaptic NMDAR causes an increase of nuclear Ca^2+^ (Wang et al., 2020b). Nuclear Ca^2+^ is one of the most effective activators of neuronal gene expression, and nuclear Ca^2+^ can regulate about 200 neuronal genes in hippocampal neurons (Zhang et al., 2007, 2009). The CREB is a signal-regulating transcription factor that plays a critical role in neuronal survival, synaptic plasticity, neurogenesis, learning, and memory. Activated NMDAR results in translocation of CREB regulators from synapse to nucleus (Hardingham and Bading, 2010). In hippocampal neurons, CREB-dependent gene expression was associated with neuroprotection against apoptosis and excitatory damage, which depends on the nuclear Ca^2+^ signaling (Papadia et al., 2005). However, some studies have also shown that activated extrasynaptic NMDARs can promote the shutdown of CREB, thereby causing mitochondrial membrane potential loss and cell death. Meanwhile, activated synaptic NMDARs activate only the CREB pathway and do not activate apoptosis (Hardingham and Bading, 2010; Franchini et al., 2020).

**Figure 3:**
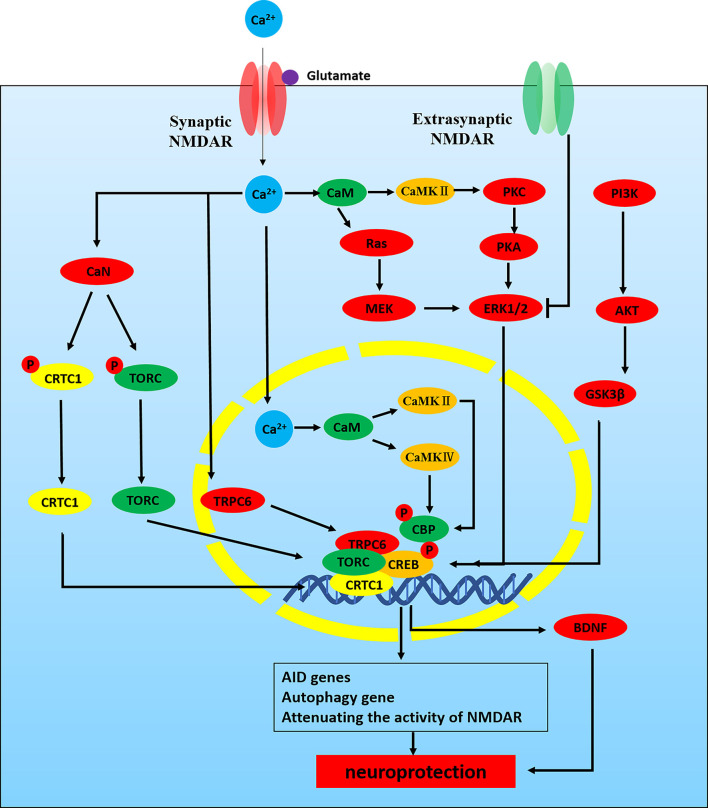
NMDAR -Ca^2+^-CREB signaling pathways in neuroprotection. NMDAR activity can activate CREB-dependent gene expression. CREB must be phosphorylated at serine-133 in order to recruit its co-activator CREB binding protein (CBP). Phosphorylation of CREB is mediated by the fast-acting nuclear Ca^2+^/CaMK pathway and the slower acting, longer lasting Ras-ERK1/2 pathway, both of which are promoted by activation of synaptic NMDARs. (1) Nuclear Ca^2+^-CaM-CaMKIV/CaMKII-CREB: nuclear Ca^2+^-dependent CaMKIV/CaMKII phosphorylates CBP at serine-301. (2) ERK1/2-CREB: CBP is also phosphorylated by Ras-MEK-ERK1/2 pathway or CaMKII/PKC/PKA-ERK1/2 pathway. CREB phosphorylated at serine-133 recruits its CBP. In addition, nuclear translocation of TORC activity is a key step in CREB activation. (3) Ca^2+^-TORC-CREB: synaptic NMDAR-induced Ca^2+^ signals promote TORC import into the nucleus by CaN-dependent dephosphorylation. TORC acts at least in part by assisting in the recruitment of CBP to CREB. (4) Ca^2+^-CRTC1-CREB: CRTC1 dephosphorylates at Ser-151 and is recruited from cytoplasm to the nucleus, where it competes with FXR for binding to CREB and drives autophagy gene expression. (5) Ca^2+^-TRPC6-CREB: Ca^2+^ influx through TRPC6 activates CREB, an important transcription factor linked to neuronal survival. (6) PI3K-AKT-GSK3β-CREB.

Synaptic NMDAR activity can activate CREB-dependent gene expression by a variety of signal pathways (Figure 3). CREB phosphorylates at serine-133 in order to recruit its co-activator CREB binding protein (CBP). Phosphorylation of CREB is mediated by the fast-acting nuclear Ca^2+^/CaMK pathway and the slower acting, longer lasting Ras-ERK1/2 pathway, both of them are promoted by activation of synaptic NMDARs (Hardingham and Bading, 2010). Nuclear Ca^2+^-dependent CaMKIV/CaMKII phosphorylates CBP at serine-301. Meanwhile, CBP is also phosphorylated by the Ras-MEK-ERK1/2 pathway or the CaMKII/PKC/PKA-ERK1/2 pathway (Cortés-Mendoza et al., 2013; Lyu et al., 2020). Nuclear translocation of the transducer of regulated CREB (TORC) activity is a key step in CREB activation. Synaptic NMDAR-induced Ca^2+^ signals promote TORC import into the nucleus by calcineurin (CaN)-dependent dephosphorylation (Screaton et al., 2004; Kovács et al., 2007). TORC also acts at least in part by assisting in the recruitment of CBP to CREB. Meanwhile, CREB-regulated transcription coactivator 1(CRTC1) can also dephosphorylate at Ser-151 and is recruited from cytoplasm to the nucleus, where it competes with FXR (fed-state sensing nuclear receptor) for binding to CREB and drives autophagy gene expression (Pan et al., 2021). Some studies have shown that Ca^2+^ influx activates CREB through TRPC6, which is an important transcription factor linked to neuronal survival. Activated TRPC6 may inhibit neuronal NMDAR activity through the post-translational means to combat glutamate-induced excitotoxic damage (Shekhar et al., 2021). Finally, CREB can also be activated through the PI3K-AKT-GSK3β pathway and play a neuroprotective role in the hippocampus. GSK-3β deletion also inhibits the activity-dependent neural activation and Ca^2+^/CaMKIV/CaMKII-CREB signaling (Liu et al., 2017; Srivastava et al., 2018). Related studies have shown that the epileptogenesis of pilocarpine-induced medial temporal lobe epilepsy (MTLE) is associated with abnormal regulation of NMDAR-mediated excitatory neuronal mechanisms and neuronal activity regulated by Ca^2+^/CaMK signaling (Canto et al., 2021).

The gene of neurotrophin BDNF is regulated by nuclear Ca^2+^-CREB signaling (Favaron et al., 1993; Hardingham and Bading, 2010). NMDAR activation increases the release of BDNF, which protects neurons from damage caused by NMDAR blockade (Fabbrin et al., 2020; Lian et al., 2021). Some studies have shown that improving mitochondrial dynamics and increasing the activity of the NMDAR-CREB-BDNF pathway could ameliorate synaptic function and neuronal survival in SAMP8 mice (Lian et al., 2021). Synaptic NMDARs and extrasynaptic NMDARs have different physiological functions. Activated synaptic NMDARs lead to phosphorylation and activation of CREB, while activated extrasynaptic NMDARs inhibit CREB pathway (Hardingham and Bading, 2010). ERK1/2 pathway also promotes CREB activation and inactivates the pre-death protein BAD, which is associated with NMDAR-dependent neuroprotection (Hetman and Kharebava, 2006). In addition, nuclear factor I subtype A (NFIA), an NMDAR-dependent activation of other neuroprotective factors, may not be associated with the increase of nuclear Ca^2+^, but its activation depends on the ERK1/2 pathway and nNOS (Zheng et al., 2010).

The NMDAR-CREB-BDNF pathway plays an important role in inhibiting epileptic seizures (Yu et al., 2019; Sharma et al., 2021). Recent studies have indicated that CREB is involved in the etiology of epilepsy (Wang G. et al., 2020). In the KA-induced epilepsy model, CREB is considered to be one of the main upstream transcription factors regulating gene expression and is closely related to the severity of epilepsy (Conte et al., 2020). Meanwhile, recent studies have shown that CREB reduces oxidative neuronal damage in TLE associated with cognitive impairment (Xing et al., 2019). In addition, microRNA-204 also inhibits the epileptiform discharge of hippocampal neurons *in vitro* by regulating TrkB-ERK1/2-CREB signaling (Xiang et al., 2016). NMDAR mediates CREB-dependent gene expression, which is closely associated with neuroprotection against apoptosis and excitatory damage, so the study of the regulatory mechanism of the NMDAR-CREB pathway in epilepsy is conducive to the neuroprotection of the epileptic brain. However, the regulatory mechanism of the NMDAR-CREB pathway in epilepsy is not entirely clear and needs further exploration.

## The Regulation of Nmdar in Epilepsy

Although NMDAR affects the occurrence of epilepsy through a variety of mechanisms, it is also regulated by a variety of factors. Activation of the NMDAR is a cooperative process, which depends on: (1) the relief of Mg^2+^ block of the ion channel pore; (2) depolarization of the postsynaptic membrane; and (3) the agonist glutamate, and co-agonists (glycine, D-serine; Jorratt et al., 2021). In addition, the expression and function of NMDAR are also affected by the expression and transcription process of related NMDAR genes, microRNAs, related proteins, and signaling pathways. On this basis, we will discuss a variety of factors for the regulation of NMDARs in the occurrence, development, and treatment of epilepsy (Tables s 1, 2).

**Table 1:** The regulation of NMDAR in epilepsy.

Factors		Mechanisms	References
NMDAR antagonists	Ketamine	Ketamine inhibited the expression of NMDAR and increased the sensitivity of neurons to excitotoxicity. Ketamine use in the treatment of RSE and SRSE.	Liu et al. (2011), Huang et al. (2012), Yan et al. (2014), Li et al. (2016, 2017), Niquet et al. (2017), Ye et al. (2018), Santoro et al. (2019), Alkhachroum et al. (2020), Borsato et al. (2020), Lu et al. (2020), Marrero-Rosado et al. (2020), Samanta (2020), Tannich et al. (2020, 2021), and Lumley et al. (2021)
	Memantine	*GRIN2A* missense mutation retained sensitivity to memantine, and memantine test results showed a significant reduction in seizure frequency. The patients with *GRIN2B* mutation-related encephalopathy treated with memantine had improved consciousness, behavior and sleep, but none showed a reduction in seizure frequency.	Pierson et al. (2014), Platzer et al. (2017), and Sun et al. (2020)
	Allosteric modulators	The latter epilepsy patients might respond to positive allosteric modulators of the NMDARs	Zhu and Paoletti (2015)
Amino acids	Glutamate	NMDAR is one of the excitatory receptors that glutamate acts on directly and may lead to diseases such as epilepsy, stroke, AD, and PD.	Alcoreza et al. (2021) and Essiz et al. (2021)
	Glycine	Glycine binds to glycine binding sites on NMDAR to regulate the function of NMDAR.	Mothet et al. (2015)
	D-serine	D-serine regulates NMDAR by binding to the receptor’s glycine binding site. The expression of D-serine and NMDAR was significantly increased in patients with intractable epilepsy. The expression of D-serine depends on the regulation of SR and DAAO.	Mothet et al. (2015), Zhu and Paoletti (2015), Ploux et al. (2020), Beesley et al. (2021), Takagi et al. (2021) and Zhang et al. (2021)
	Cysteine/Homocysteine (HCY)	Redox modulation of cysteine residues is one of the post-translational modifications of NMDAR. HCY activates GluN2 subunit-dependent redox regulation of NMDAR by the reduction of NMDAR disulfide.	Kim et al. (2017), Ivanova M. et al. (2020), and Sibarov et al. (2020)
Magnesium (Mg)		NMDAR channel is blocked by Mg^2+^ at neuronal resting membrane potential, and Mg^2+^ is removed when the membrane is depolarized. Magnesium sulfate can inhibit glutamatergic signaling, thereby altering Ca^2+^ influx, leading to reduced excitotoxicity. TLE cell model is often established by magnesium-free extracellular fluid. Transient culture of hippocampal neurons in magnesium-free induces rhythmic and synchronous epileptiform-like activity.	Mayer et al. (1984), Wang et al. (2020b), Elsayed et al. (2021), Jorratt et al. (2021), Li et al. (2021), Mele et al. (2021), Nikolaev et al. (2021), and Zhou et al. (2021)
The EphB-NMDAR interaction		In epilepsy, the interaction of NMDAR-EphB2 was found in anti-NMDAR encephalitis.	Dalva et al. (2000), Henderson et al. (2001), Hughes et al. (2010), Nolt et al. (2011), Gleichman et al. (2012), Mikasova et al. (2012), Geng et al. (2013), Planagumà et al. (2016), Hu et al. (2017), Ernst et al. (2019), Wang et al. (2020a), Washburn et al. (2020), and Ma et al. (2021)
Epigenomics		DNMT3A1 is controlled by activated NMDAR and the expression of NMDAR is also mediated by epigenomics. In epilepsy, *GRIN2B* DNA methylation levels were increased and *BDNF* DNA methylation levels were decreased, which leading to decreased mRNA and protein expression of GluN2B and increased mRNA and protein expression of BDNF. Suppressive DNMT can increase excitatory postsynaptic potential in hippocampal slices of epileptic rats. Increased TBR1 expression in AF9 mutants is associated with increased expression of GluN1 subunit which is regulated by TBR1.	Büttner et al. (2010), Jiang et al. (2010), D’Aiuto et al. (2011), Ryley Parrish et al. (2013), Kiese et al. (2017), Fachim et al. (2019), Li et al. (2019), Bayraktar et al. (2020), and de Sousa Maciel et al. (2020)
Proteins and signaling pathways	SPARCL-1	SPARCL-1 localizes to excitatory synapses after SE; SPARCL-1 is involved in synaptic modifications underlying epileptogenesis and remodeling events associated with neuronal degeneration following neural injury.	Chen et al. (2020b) and Gan and Südhof (2020)
	SPDI	SPDI knockdown inhibit seizure activity by nitrososylation-independent thiolation on NMDAR in acute and chronic epileptic model.	Jeon and Kim (2018)
	POSH	POSH is involved in epilepsy by increasing surface NMDAR expression.	Wang X. et al. (2017)
	Nwd1	Inhibition of Nwd1 activity can reduce the hyperexcitability and GluN2B phosphorylation of hippocampal neurons.	Yang et al. (2019)
	TMEM25	TMEM25 modulates the degradation of GluN2B subunits and neuronal excitability.	Zhang et al. (2019)
	DAPK1	DAPK1 interacts with NMDAR and involves in glutamate-induced neurological events, such as stroke. Inhibiting DAPK1 can lead to phosphorylation and surface normalization of GluN2B expression outside the synapse.	DeGregorio-Rocasolano et al. (2020), Schmidt et al. (2020), and Liu et al. (2021)
	PDI	PDI binds to NMDAR in chronic epileptic rats and increases the mercaptan content on recombinant GluN1. PDI can catalyze disulfide bond formation, reduction, and isomerization.	Kim et al. (2017)
	CyclinB/CDK1	CyclinB/CDK1 mediates NMDAR phosphorylation and regulates calcium kinetics and mitosis.	Rosendo-Pineda et al. (2020)
	NSPA	NSPA regulates the postsynaptic stability of NMDAR by ubiquitination of tyrosine phosphatase PTPMEG.	Espinoza et al. (2020)
	SULT4A1	SULT4A1 promotes the formation of PSD-95/NMDAR complex to modulate synaptic development and function.	Culotta et al. (2020)
	PCDH7	PCDH7 interacts with GluN1 subunit to regulate the dendritic spine morphology and synaptic function.	Wang Y. et al. (2020)
	Leptin	Leptin resists to glutamate-induced excitotoxicity in HT22 hippocampal neurons and leptin also increases postsynaptic NMDAR currents to sensitize NTS neurons to vagal input	Jin et al. (2018) and Neyens et al. (2020)
	P2X2 and P2X4	Both P2X2 and P2X4 interact with NMDAR in an inhibitory manner.	Rodriguez et al. (2020)
	NRG1-ErbB4 signaling	NRG1-ErbB4 signaling inhibits phosphorylation of GluN2B at position 1472 by Src kinase. NRG1-ErbB4 signaling may act as a homeostasis regulator, which can protect the brain from the seizure-like activity aggravation.	Zhu et al. (2017)
	ERK1/2 signals	CCL2 rapidly enhances NMDA-induced neuronal electrical currents through the ERK-Glun2B pathway. CXCR7 regulates GluN2A expression by activating ERK1/2, thereby modulating NMDAR-mediated synaptic neurotransmission in hippocampal granulosa cells. Icaritin (ICT) has a neuroprotective effect on glutamate-induced neuronal damage and its mechanism may be associated with inactivating GluN2B-containing NMDAR by ERK/DAPK1 pathway.	Xu T. et al. (2019), Zhang H. et al. (2020), and Liu et al. (2021)
	Cholinergic signals	ACh potentiates NMDARs through muscarinic receptors in CA1 neurons of the hippocampus. Nicotinic α7-nAChR is enriched in the glutamate network synapses in the dorsolateral PFC (dlPFC) and is required for NMDAR action.	Markram and Segal (1990), Flores-Hernandez et al. (2009), and Yang et al. (2013).
	Redox modulation	Cysteine, HCY and PDI are involved in redox modulation of NMDAR. H_2_S blocks the enhancement of neuronal excitability in the early hippocampal network by inhibiting voltage-gated sodium channels and NMDARs.	Kim et al. (2017), Yakovlev et al. (2017), Ivanova M. et al. (2020), and Sibarov et al. (2020)
β-hydroxybutyrate and acetone		The inhibitory effect of β-hydroxybutyrate and acetone in NMDARs may be the basis for the therapeutic benefits of ketogenic diet in epilepsy.	Pflanz et al. (2019)

**Table 2:** Regulation of microRNAs on NMDARs in nervous system.

MicroRNAs	Mechanisms	References
MicroRNA-219, MicroRNA-219a-2	MicroRNA-219 has a regulatory effect on NMDAR in the amygdala and hippocampus of patients with mesial TLE microRNA-219 protects against seizure in the KA-induced epilepsy model. MicroRNA-219a-2 can reduce calcium overload and apoptosis by HIF1α/NMDAR pathway.	Zheng et al. (2016), Hamamoto et al. (2020), and Hu et al. (2020)
microRNA-139-5P	MicroRNA-139-5P has a negative regulatory effect on GluN2A-NMDAR in pilocarpine-induced epilepsy model and TLE patients.	Alsharafi et al. (2016)
MicroRNA-34c	MicroRNA-34c plays a negative role in epileptic seizure cognitive function, by regulating NMDARs and AMPARs associated with LTP.	Huang et al. (2018)
microRNA-15a-5p	Both in hippocampal tissues of SE rats and low Mg-induced hippocampal neurons, propofol can inhibit apoptosis of hippocampal neurons by microRNA-15a-5p/GluN2B/ERK1/2 pathway	Liu et al. (2020)
MicroRNA-124	MicroRNA-124 suppresses seizure and regulates CREB1 activity. Inhibition of neuronal firing by microRNA-124 is associated with the suppression of AMPAR- and NMDAR-mediated currents, accompanied by decreased expression of NMDAR	Wang et al. (2016)
MicroRNA-211, microRNA-128	microRNA-211 or microRNA-128 transgenic mice displayed seizures.	Feng et al. (2020)
MicroRNA-223	MicroRNA-223 regulates the expression of GluN2B subunit, plays a therapeutic role in stroke and other excitotoxic neuronal disorders.	Harraz et al. (2012)
MicroRNA-132, microRNA-107	MicroRNA-132 and microRNA-107 could involve in NMDAR signaling by influencing the expression of pathway genes or the signaling transmission.	Zhang et al. (2015)
MicroRNA-19a, microRNA-539	MicroRNA-19a and microRNA-539 can influence the levels of NMDARs subunits by targeting the mRNAs encoding GluN2A and GluN2B subunits respectively.	Corbel et al. (2015)
MicroRNA-125, microRNA-132	FMRP is an RNA-binding protein responsible for interacting with microRNA-125 and microRNA-132 to regulate NMDAR, and consequently affecting synaptic plasticity	Lin (2015)
MicroRNA-204	EphB2 is a direct target of microRNA-204 and microRNA-204 downregulates EphB2 in hippocampal neurons. EphB2 regulates the surface expression of the NMDAR GluN1 subunit.	Mohammed et al. (2016)
MicroRNA-182-5p	MicroRNA-182-5p regulates nerve injury-induced nociceptive hypersensitivity by targeting EphB1 which interacts with the NMDAR	Zhou et al. (2017)

### NMDAR Antagonists

NMDAR antagonists play an important role in the treatment of epilepsy. NMDAR antagonists enhance the anticonvulsant effect of lithium chloride on PTZ-induced clonic seizures in mouse (Ghasemi et al., 2010). Animal studies have suggested NMDAR antagonists may become more effective with seizures lasting longer after the failure of the first line therapies (Sánchez Fernández et al., 2019). In addition, the latter epilepsy patients might respond to positive allosteric modulators of the NMDARs (Zhu and Paoletti, 2015).

#### Ketamine

Ketamine is a noncompetitive NMDAR antagonist and blocks Ca^2+^ influx by binding to phencyclidine-binding sites of NMDAR. Meanwhile, it is used for evidence of clinically RSE and SRSE (Borsato et al., 2020). Ketamine induces developmental neurotoxicity by inhibiting the expression of NMDAR and increasing the sensitivity of neurons to glutamate excitotoxicity, thereby leading to deregulation of Ca^2+^ signaling and triggering oxidative stress and even mitochondrial apoptosis pathways in neurons. Ketamine significantly upregulates the GluN1 subunit of NMDAR in the frontal cortex, thereby triggering neuronal apoptosis (Liu et al., 2011). Meanwhile, mitochondrial dysfunction and oxidative stress in the hippocampus of rats exposed to ketamine are associated with down-regulation of the ERK signaling cascade (Huang et al., 2012; Li et al., 2017). In addition, ketamine induces apoptosis through the mechanism associated with caspase-1-dependent pyroptosis in the hippocampus (Ye et al., 2018). Ketamine also aggravated cognitive impairment and hippocampal neurodegeneration through the ROS/HIF-1α pathway (Yan et al., 2014). However, ketamine inhibits lipopolysaccharide-mediated BV2 microglia inflammation by blocking NMDARs (Lu et al., 2020). It has been shown that ketamine inhibits the NOX2 activation to produce ROS of the mice brain in pilocarpine-induced epilepsy (Tannich et al., 2021). Ketamine-midazolam therapy can reduce the severity of seizures and improve brain pathology in plasma carboxylesterase knockout mice (Marrero-Rosado et al., 2020). A low dose of ketamine can reduce the behavioral changes in pilocarpine-induced epilepsy mice (Tannich et al., 2020).

In clinical studies, ketamine was used in the treatment of RSE and SRSE (Samanta, 2020). Ketamine treatment is associated with a decrease in seizure burden in patients with SRSE (Alkhachroum et al., 2020). Midazolam-ketamine-valproate therapy is significantly more effective than midazolam-fosphenytoin-valproate therapy in seizure reduction (Niquet et al., 2017). Meanwhile, the combination of ketamine-midazolam can reduce the severity of epilepsy, epileptogenesis, and neuropathology in cholinergic-induced SE (Lumley et al., 2021). And ketamine is also used in anti-NMDAR encephalitis-associated RSE (Santoro et al., 2019). Severe epileptic encephalopathy caused by *GRIN2D* mutations can be treated with NMDAR channel blockers (ketamine, magnesium; Li et al., 2016).

#### Memantine

Different NMDAR subunit gene mutations also have different responses to NMDAR antagonists. it was reported that a 9-year-old boy with severe early-onset epileptic encephalopathy caused by a GRIN2A missense mutation was trialed on memantine and a significant reduction in seizure frequency was revealed (Pierson et al., 2014). Whereas, another study showed that no seizure reduction was found in patients with *GRIN2B* mutation-related encephalopathy treated by memantine despite improved consciousness, behavior, and sleep (Platzer et al., 2017). In addition, a combination of memantine and cathodal direct current stimulation (cDCS) suppressed KA-induced seizures (Sun et al., 2020). Thus, early identifying the location and type of NMDAR subunit gene mutation in epilepsy has guiding significance for the selection of AEDs.

### Magnesium (Mg)

The NMDAR ion channel pores are permeable to Ca^2+^ but can be blocked by Magnesium ion (Mg^2+^) in a strongly voltage-dependent manner, which makes them largely inactive at resting voltages, even in the presence of agonists (Mayer et al., 1984; Nikolaev et al., 2021). Activation of the NMDAR is a cooperative process, which depends on the relief of the Mg^2+^ block of the ion channel pore (Mg^2+^ is removed into the extracellular compartment from the channel pore; Hou et al., 2020; Jorratt et al., 2021). The NMDAR channel is blocked by Mg^2+^ at neuronal resting membrane potential, and Mg^2+^ is removed when the membrane is depolarized (Jorratt et al., 2021; Li et al., 2021). In addition, non-competitive NMDAR ion channel blockers such as MK-801 mainly bind to ion channels of TMD. Since Mg^2+^ normally blocks this channel, the binding of MK-801 requires NMDAR activation and depolarization to release Mg^2+^ (Wong et al., 2021). A relevant study has shown that Mg^2+^ influx, dependent on NMDAR opening, can transduce a signaling pathway to activate CREB in neurons (Hou et al., 2020). Magnesium sulfate is a neuroprotective agent in clinical practice. By noncompetitively blocking NMDARs, magnesium sulfate can inhibit glutamatergic signaling, thereby altering Ca^2+^ influx, leading to reduced excitotoxicity (Elsayed et al., 2021). *In vitro*, TLE cell model is often established by treating primary hippocampal cells with magnesium-free extracellular fluid. Transient culture of hippocampal neurons in magnesium-free induces rhythmic and synchronous epileptiform-like activity (Mele et al., 2021).

The low-affinity binding site of Mg^2+^ is located deep in the ion channel and is modulated by the NMDAR subunit. Related studies have shown that NMDAR complexes formed by GluN2A or GluN2B subunits have a higher affinity for Mg^2+^ than those containing GluN2C or GluN2D (Monyer et al., 1994). Due to different GluN2 subunits, NMDAR has different sensitivity to Mg^2+^ (Valdivielso et al., 2020). An important feature of the GluN2 subunits is that GluN2A and GluN2B subunits are more sensitive to voltage-dependent Mg^2+^ blocking than GluN2C and GluN2D subunits (Qian et al., 2005). Meanwhile, the GluN2C subunit contributes to a lower threshold for Mg^2+^ block and influences NMDAR agonist activity (Intson et al., 2020). Compared to GluN1/GluN2D receptors or other NMDAR subtypes, GluN1/GluN2C receptors exhibit higher blockade with ketamine in the presence of Mg^2+^(Shelkar et al., 2019). The human NMDAR GluN2A variant influences channel blocker potency. A novel genetic variant of *GRIN2A* has been identified in patients with epileptic encephalopathy altering residues located in the NMDAR ion channel pore and significantly reducing Mg^2+^ blockade and channel conductance (Marwick et al., 2019). In functional studies, the *GRIN2A* mutation decreased the potency of endogenous negative modulators, including magnesium and zinc (Fernández-Marmiesse et al., 2018). In addition, missense mutations of *GRIN2B* also alter NMDAR ligand binding and ion channel properties. *GRIN2B* mutants showed decreased glutamate potency, increased NMDAR desensitization, and disappearance of voltage-dependent Mg^2+^ block (Fedele et al., 2018). Meanwhile, Mg^2+^ deficiency down-regulated GluN2B subunits expression in cultured hippocampal slices (Zhou et al., 2021). In addition, presynaptic release and postsynaptic transporter transport zinc (Zn) to different microdomains to regulate NMDAR neurotransmission. Meanwhile, zinc inhibits synaptic NMDARs, which depend on the binding of GluN2A to zinc transporter ZnT1(Krall et al., 2020). In conclusion, Mg^2+^ plays an important role in the pathogenesis of epilepsy. The most prominent of these is voltage-dependent block of the NMDAR channel by Mg^2+^.

### Amino Acids

#### Glutamate

**Figure 4:**
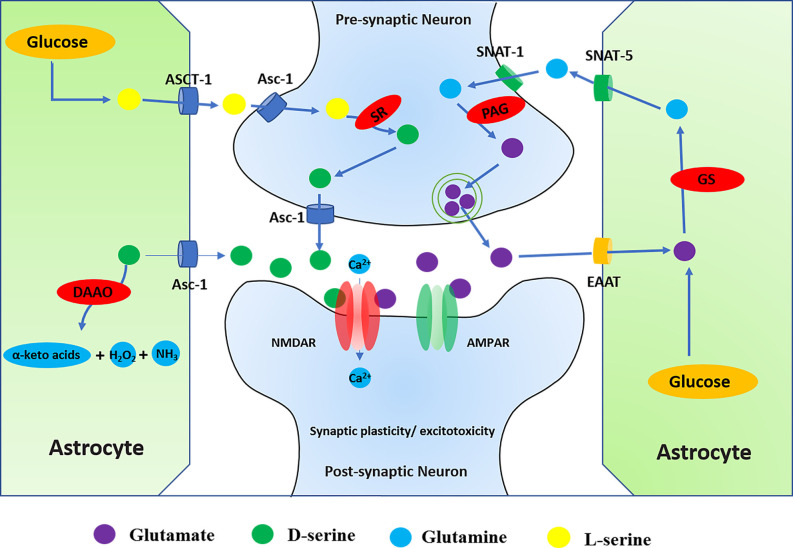
Regulation of NMDARs by D-serine and glutamate. (1) Glutamate can directly act on NMDAR, and the glutamate-glutamine cycle is involved in the regulation of NMDAR. Glutamate-glutamine cycle: glutamate can be directly synthesized *de novo* by astrocytes or indirectly produced from glucose molecules through the actions of pyruvate dehydrogenase and astrocyte-specific enzyme pyruvate carboxylase in the brain. Meanwhile, extracellular glutamate can be transferred to astrocytes by ETTA2 (GLT-1) and then converted to glutamine by glutamine synthetase (GS). Glutamine is transported by SNAT-5 to the extracellular environment, where it can then be transferred to neurons by SNAT-1. In the neuron, glutamine is degraded by PAG into glutamate and ammonia. Glutamate enters the synaptic vesicles in the pre-synaptic neurons and then is released from the pre-synaptic membrane into the synaptic cleave. It directly acts on the NMDAR in the post-synaptic neurons, thus activating NMDAR. (2) In addition to glutamate, activation of NMDAR also requires the binding of D-serine at the glycine binding site. SR converts L-serine to D-serine in the neuron, while DAAO catalyzes the breakdown of d-serine in the astrocyte. D-serine is released from neurons by Asc-1, which mediates D-serine efflux in exchange for external amino acid substrates. L-serine can be directly synthesized *de novo* in astrocytes. Through orchestrated Asc-1 and ASCT1 subtypes, L-serine from astrocytes enters the neuron and is catalyzed by SR to produce D-serine.

It is widely believed that the imbalance between excitatory and inhibitory neurotransmission leads to hyperexcitability of neuronal circuits, which is the basis of the process of epileptogenesis (Alcoreza et al., 2021). Glutamate is an excitatory neurotransmitter in the brain involved in various neural functions and metabolic processes of the CNS (Wang et al., 2020b). NMDAR is one of the excitatory receptors that glutamate acts on directly and may lead to diseases such as epilepsy, stroke, AD, and PD (Essiz et al., 2021). On the one hand, glutamate can be directly synthesized *de novo* by astrocytes in the brain. On the other hand, glutamate can also be produced indirectly with glucose molecules through the action of pyruvate dehydrogenase and pyruvate carboxylase in astrocytes (Schousboe et al., 2014). Meanwhile, extracellular glutamate can be transferred to astrocytes by excitatory amino acid transporter 2 (EAAT2) and then converted to glutamine by glutamine synthetase (GS). Glutamine is transported by astrocytic glutamine transporter-5 (SNAT-5) to the extracellular environment, where it can then be transferred to neurons by astrocytic glutamine transporter-1 (SNAT-1; Danbolt, 2001). In the pre-synaptic neurons, phosphate-activated glutaminase (PAG/GLS-1) converts inactive glutamine to glutamate, which is repackaged into synaptic vesicles and released into the synaptic cleft and directly acts on the NMDAR in the post-synaptic neurons, thus activating NMDAR (Limón et al., 2021). In a word, the glutamate and glutamine cycle in astrocytes and neurons is called the glutamate-glutamine cycle (Figure 4).

Astrocyte dysfunction can alter glutamate homeostasis, leading to neuroexcitatory toxicity (Niciu et al., 2012). Excessive glutamate can cause neuroexcitatory toxicity after being released into the synaptic cleft, and excess glutamate needs to be cleared quickly in the brain (Schousboe et al., 2014). However, due to the absence of extracellular enzymes, the uptake of extracellular glutamate mainly relies on EAATs which are located in the plasma membranes of neurons and glia (Zhang et al., 2016). Meanwhile, astrocytes can also completely enclose the glutamate synapse to quickly clear glutamate from the synaptic cleft (Alcoreza et al., 2021). Once glutamate enters the astrocyte, it is converted to glutamine and returned to the neuron by the glutamate-glutamine cycle (Alcoreza et al., 2021). In addition, over-activated astrocytic NMDARs could lead to the release of many other molecules that are likely to be relevant. Astrocyte GluN2A regulates nerve growth factor β (β-NGF) synthesis, maturation, and secretion by regulating pNF-κB, Furin, and VAMP3.

It is found that the neuroprotective role of astrocytic GluN2A in the promotion of synapse survival is by regulating these molecules (Du et al., 2021). Both glutamate and quinolinic acid (QUIN) could activate astrocytic NMDARs, which stimulate Ca^2+^ influx into the cell and can result in dysfunction and death of astrocytes (Lee et al., 2010).

#### D-Serine

In addition to glutamate, the activation of NMDAR also requires the binding of a co-agonist at the glycine binding site. Originally similar to glycine, D-serine can also control the activation of NMDAR by binding to the receptor’s glycine binding site in the brain (Figure 4; Mothet et al., 2015). Recent studies have indicated that the expression of D-serine and NMDAR is closely related to intractable epilepsy (Zhu and Paoletti, 2015). The review investigated the regulation of NMDAR by D-serine in CNS diseases, including epilepsy.

D-serine is the main endogenous co-agonist of NMDARs, which is significantly dependent on the activity of the metabolic enzyme d-amino acid oxidase (DAAO) and serine racemase (SR). In the brain, DAAO catalyzes the decomposition of D-serine, while the cytoplasmic enzyme SR converts L-serine to D-serine (Takagi et al., 2021). SR is mainly found in neurons, that catalyzes the reversible racemization of L-serine and D-serine (Raboni et al., 2019). On the one hand, D-serine is released from the neuron *via* a plasma membrane transporter (ASC-1/SLC7A10), which mediates D-serine efflux. On the other hand, astrocytes synthesize SR substrate L-serine, which is transferred to neurons through a mechanism of serine shuttle to participate in neuronal D-serine synthesis. L-serine synthesized by astrocytes is dependent on the activity of 3-phosphoglycerate dehydrogenase (PHGDH), which is the key to *de novo* synthesis of L-serine and activation of NMDAR (Neame et al., 2019). Clinically, serine deficiency patients present with severe neurological symptoms, including intractable epilepsy, which suggests the relevance of serine to brain development and morphogenesis (Murtas et al., 2020). Although the activity of SR and DAAO is important for the regulation of D-serine levels, the regulatory mechanisms of SR and DAAO are not fully understood in epilepsy.

DAAO selectively catalyzes the oxidative deamination of natural D-serine to produce imino acid, which is naturally hydrolyzed to the corresponding α-keto acids and ammonia (Pollegioni et al., 2018). Related research has confirmed that inhibition of DAAO can lead to increased D-serine in the brain, thereby regulating a variety of neurophysiological functions including cognitive behavior (Nagy et al., 2020). Meanwhile, it was also found that NMDAR antagonists (MK801 and cocaine) could increase the release of glutamate and decrease the expression of SR and DAAO. However, D-serine and antipsychotics did not modulate the levels of SR and DAAO (Takagi et al., 2021). In addition, the neuroprotective effect of DAAO is also mediated by the ERK1/2 signal pathway (Zhang X. et al., 2020).

In the brain, SR is mainly in excitatory neurons and GABAergic inhibitory interneurons, and is only weakly expressed in astrocytes (Billard, 2018). Through the coordinated activities of ASC-1 and ASCT1 subtypes, D-serine is released and binds with NMDAR to perform neurophysiological functions (Sason et al., 2017; Billard, 2018; Kaplan et al., 2018). The deletion of SR affects the balance of excitatory and inhibitory in the hippocampal CA1 network (Ploux et al., 2020). In addition, related research has found that PKC phosphorylates SR on serine residues and reduces the activity of SR *in vitro*. Similarly, activated PKC also increases SR phosphorylation and decreases the levels of D-serine in the rat frontal cortex (Vargas-Lopes et al., 2011). Therefore, PKC-mediated SR phosphorylation may be important for the activation of NMDARs.

In fact, the specific degradation of D-serine by the enzyme DAAO and the genetic deletion of SR significantly altered the activation of NMDAR. D-serine plays a key role in regulating the functional plasticity of many synapses in the brain (Billard, 2018). Related studies have found that the beneficial effects of D-serine supplementation may reflect that D-serine levels decreased significantly with age, as supported in the hippocampal trial. Interestingly, this decline of D-serine is also found in human plasma levels (Potier et al., 2010). In addition, in addition to affecting synaptic plasticity and synaptogenesis, dysregulation of D-serine metabolism may also enhance NMDAR-dependent excitotoxicity and promote cognitive impairment and neurodegeneration (Beltrán-Castillo et al., 2018). Therefore, the synaptic availability of D-serine and the preservation of SR activity are critical for maintaining strong cognitive abilities in the brain.

D-serine and NMDAR were found to be significantly upregulated in patients with intractable epilepsy (Zhang et al., 2021). Therefore, the D-serine signal pathway may be a potential target for epilepsy therapy. Endogenous D-serine deficiency may lead to decreased inhibition of the hippocampal CA1 network and altered excitatory/inhibitory balance. besides, D-serine contributes to maintaining cognitive abilities and functional plasticity of synapses (Ploux et al., 2020). Related studies have shown that intracranial injection of D-serine into the medial entorhinal area (MEA) in the TLE is beneficial to prevent neuronal loss and epileptogenesis by rescuing hippocampal CA1 neurons in the epileptic brain and reducing the number of astrocytes and microglia, thus alleviating the effect of neuroinflammation (Beesley et al., 2021). Therefore, D-serine might be a potential therapy target *via* regulating NMDAR in epilepsy, and more studies are needed in the future.

### Epigenomics

DNA methylation is a crucial epigenetic mark for activity-dependent gene expression in neurons. It has been shown that the levels of DNA methyltransferase 3A1 (DNMT3A1) in neurons are closely controlled by the activation of NMDAR-containing GluN2A subunits (Bayraktar et al., 2020). Interestingly, synaptic NMDARs drive methyltransferase degradation in a ubiquitin-like dependent manner. The binding of NEDD8 ubiquitin-like protein to lysine residues inhibits ubiquitination, thereby blocking DNMT3A1 degradation (Bayraktar et al., 2020). Defects in promoter methylation of these activity-dependent genes may be related to synaptic plasticity and memory formation (Bayraktar et al., 2020). Overall, the activity-dependent DNA methylation is regulated by GluN2A-containing NMDAR signals to participate in memory formation. Meanwhile, NO produced by NMDAR activation can also up-regulated DNA methyltransferase 3B (DNMT3B) in the hippocampus (de Sousa Maciel et al., 2020). A related analysis showed that both GluN2B expression levels and histone H3K9 acetylation of *GRIN2B* gene promoter were positively correlated with ethanol withdrawal syndrome (EWS; Li et al., 2019). In addition, the changes of *GRIN2B* promoter methylation may be associated with cognition reduction and glutamatergic dysfunction in schizophrenia (Fachim et al., 2019). Meanwhile, this epigenetic change leads to upregulation of functional NMDAR and abnormal neuronal differentiation (D’Aiuto et al., 2011). In addition, NMDAR expression is also regulated by histone methylation in the brain. Histone methyltransferases targeting histone H3-lysine 9 residues, including Setdb1 (Set domain, bifurcated1)/Eset/Kmt1e, are closely related to inhibition of chromatin remodeling in the adult brain. Meanwhile, the inhibition of Setdb1-mediated histone methylation at *GRIN2B* is associated with decreased expression of GluN2B (Jiang et al., 2010).

In epilepsy, control of epigenetic of epilepsy target genes contributes to cellular memory of epileptogenesis in cultured rat hippocampal neurons (Kiese et al., 2017). In cultured neurons, the altered gene expression and epigenetic modifications can be rescued by blocking action potential propagation or inhibiting glutamatergic activation (Kiese et al., 2017). The epigenetic modification of epileptic target genes and cellular memory of epileptogenesis, which can transform normal neurons and circuits into pro-epileptic neurons and neural circuits (Kiese et al., 2017). SE can also lead to abnormal expression of *GRIN2B* and *BDNF* genes in the hippocampus in TLE (Ryley Parrish et al., 2013). In the epileptic hippocampus, *GRIN2B* DNA methylation levels were increased and *BDNF* DNA methylation levels were decreased, which led to decreased mRNA and protein expression of GluN2B and increased mRNA and protein expression of BDNF. Meanwhile, suppressive DNMT can increase excitatory postsynaptic potential in hippocampal slices of epileptic rats (Ryley Parrish et al., 2013). Therefore, GluN2B DNA methylation may be an early SE-induced event that persists into late epilepsy in the hippocampus and promotes changes of gene expression in TLE. In addition, leukemia-related AF9/MLLT3 mutations are involved in neurodevelopmental disorders such as epilepsy and ataxia (Büttner et al., 2010). Meanwhile, AF9 is found to be an active epigenetic modifier by increasing the expression of GluN1 subunits during the generation of cortical projection neurons (Büttner et al., 2010).

In conclusion, the epigenetic regulation of NMDAR and epilepsy target genes by NMDAR play an important role in the onset and development of seizures. Whereas, the mechanism remains unclear and more studies are urgently needed.

### MicroRNA and NMDAR

In recent years, researchers have found a link between microRNAs and NMDARs-mediated neurological diseases (Table 2; Alsharafi et al., 2017). A growing body of evidence indicates that microRNAs regulate synaptic homeostasis and plasticity processes, suggesting that they may be involved in synaptic dysfunction during epilepsy, stroke, and AD (Alsharafi et al., 2017). MicroRNA-34a deficiency promotes cognitive function by regulating AMPARs and NMDARs to increase synaptic plasticity (Xu et al., 2018). MicroRNA-219-5p alleviates morphine tolerance by inhibiting the CaMKII/NMDAR pathway (Wang J. et al., 2017). Meanwhile, microRNA-219a-2 has been reported to reduce calcium overload and apoptosis through HIF1α/NMDAR pathway, thus alleviating myocardial ischemia-reperfusion injury (Hu et al., 2020). MicroRNA-182-5p regulates nerve injury-induced nociceptive hypersensitivity by targeting Ephrin type-b receptor 1 (EphB1) which interacts with the NMDAR (Zhou et al., 2017). EphB2 is a direct target of microRNA-204 and microRNA-204 downregulates EphB2 in hippocampal neurons. EphB2 is a known regulator of synaptic plasticity and regulates the surface expression of the NMDAR GluN1 subunit (Mohammed et al., 2016). Thus, microRNA-204 may play an important role in anti-NMDAR encephalitis by regulating EphB2-NMDAR, which remains to be explored. In normal neuronal development, FMRP is an RNA-binding protein responsible for interacting with microRNA-125 and microRNA-132 to regulate NMDAR and consequently affecting synaptic plasticity (Lin, 2015). MicroRNA-19a and microRNA-539 can influence the levels of NMDARs subunits by targeting the mRNAs encoding GluN2A and GluN2B subunits respectively (Corbel et al., 2015). MicroRNA-219, microRNA-132, and microRNA-107 could be involved in NMDAR signaling by influencing the expression of pathway genes or the signaling transmission (Zhang et al., 2015). MicroRNA-223 as a major regulator of the expression of the GluN2B subunit, plays a therapeutic role in stroke and other excitotoxic neuronal disorders (Harraz et al., 2012). These microRNAs provide an entry point for affecting neural plasticity and abnormal nerve firing and provide a new approach for the treatment of NMDAR-related neurological diseases.

In epilepsy, some microRNAs (microRNA-34, microRNA-124, microRNA-146a, microRNA-135a, microRNA-23a, microRNA-132, microRNA-234-5p, microRNA-203, microRNA-181b, microRNA-155 microRNA-219, microRNA-211, microRNA-128, microRNA-23) have been reported and each microRNA has limitations as a potential epilepsy target (Feng et al., 2020). Importantly, microRNA-211 or microRNA-128 transgenic mice displayed seizures (Feng et al., 2020). However, some microRNAs play an important role in epilepsy by regulating NMDARs. MicroRNA-219 had a regulatory effect on NMDAR in the amygdala and hippocampus of patients with mesial TLE and microRNA-219 protects against seizure in the KA-induced epilepsy model (Zheng et al., 2016; Hamamoto et al., 2020). Meanwhile, microRNA-139-5P has a negative regulatory effect on GluN2A-NMDAR in pilocarpine-induced epileptic rat models and TLE patients (Alsharafi et al., 2016). MicroRNA-34c has also been found to play a negative role in seizure and cognitive function, possibly by regulating NMDARs and AMPARs associated with LTP (Huang et al., 2018). Both in hippocampal tissues of SE rats and low Mg-induced hippocampal neurons, propofol can inhibit apoptosis of hippocampal neurons by microRNA-15a-5p/GluN2B/ERK1/2 pathway, which provides theoretical support for propofol treatment of SE (Liu et al., 2020). MicroRNA-124 suppresses seizure and regulates CREB1 activity. Inhibition of neuronal firing by microRNA-124 is associated with the suppression of mEPSC, AMPAR- and NMDAR-mediated currents, which are accompanied by decreased surface expression of NMDAR (Wang et al., 2016). However, many microRNAs have not been confirmed to function in epilepsy by regulating NMDAR. The discovery of various microRNA is also beneficial for the treatment of epilepsy and reducing the occurrence of epilepsy.

### The EphB2-NMDAR Interaction

The interaction between NMDAR and EphB2 was found in anti-NMDAR encephalitis (Hughes et al., 2010; Mikasova et al., 2012). It is reported that transcranial direct current stimulation promotes hippocampal neurogenesis in mice with cerebral ischemia by activation of Ephrinb1/EphB2/MAP-2/NMDAR pathway (Ma et al., 2021). Meanwhile, activated EphB receptors promote the excitability of primary sensory neurons either directly through Ca^2+^ influx or by phosphorylation of Src kinase-mediated NMDAR (Washburn et al., 2020). In the acute phase of ischemic stroke, EphB2-dependent signal pathways are found to promote neuronal NMDAR-induced excitotoxicity and inflammation (Ernst et al., 2019). In AD models, overexpression of EphB2 in hippocampal neurons improved impaired NMDAR and cognitive dysfunction (Hu et al., 2017). In addition, EphB2 has a positive protective effect on Aβ1-42 oligomer-induced neurotoxicity by synaptic NMDAR signal pathway in hippocampal neurons (Geng et al., 2013). EphB2 can also prevent the effects of NMDAR antibodies on memory and neuroplasticity (Planagumà et al., 2016). EphB2 regulates NMDAR function and synaptic targeting. In mature neurons, EphB2 regulates the number of synaptic NMDAR, while activated EphB2 reduces desensitization of Ca^2+^-dependent NMDAR and is required for enhanced synaptic localization of GluN2B-containing NMDAR. EphB knockout mice showed the homeostatic upregulation of NMDAR expression (Nolt et al., 2011). Synaptic plasticity is regulated by the EphB2-GluN2A-AKT cascade, which might be a potential pathogenesis of depression and potential therapeutic target of glutamatergic transmission dysfunction (Wu et al., 2019). Meanwhile, EphrinB/EphB signaling is conducive to synaptic plasticity by GluN2B phosphorylation in chronic migraine (Wang et al., 2020a). In dentate granular neurons of EphB2-deficient mouse, synaptic NMDAR-mediated current was reduced (Henderson et al., 2001). These findings suggest that EphB is a key regulator of NMDAR synaptic localization and NMDAR-dependent synaptic function in the CNS. Together, the regulation of synaptic function may be closely related to EphB2-NMDAR interaction in epilepsy.

### Influence of Related Proteins and Signaling Pathways on NMDAR

In addition to the above regulation mechanisms, NMDAR is also modulated by other pathways. Related studies have demonstrated that both purinergic P2X receptors (P2X2) and P2X4 interact with NMDAR in an inhibitory manner (Rodriguez et al., 2020). Meanwhile, SULT4A1 promotes the formation of the PSD-95/NMDAR complex to modulate synaptic development and function (Culotta et al., 2020). It is also found that S-PrP interacts with LRP1/NMDAR system to activate ERK1/2, thereby promoting cell migration in Schwann cells (Mantuano et al., 2020). Neuronal surface P antigen (NSPA) regulates the postsynaptic stability of NMDAR by ubiquitination of tyrosine phosphatase PTPMEG (Espinoza et al., 2020). In addition, Cyclin B/CDK1 mediates NMDAR phosphorylation and regulates calcium kinetics and mitosis (Rosendo-Pineda et al., 2020). Neuroinflammation modulation is known to be controlled by cholinergic signals (Mizrachi et al., 2021). However, ACh potentiates NMDARs through muscarinic receptors in CA1 neurons of the hippocampus (Markram and Segal, 1990). Nicotinic α7-nAChR is enriched in the glutamate network synapses in the dorsolateral PFC (dlPFC) and is required for NMDAR action (Yang et al., 2013). LTP can be induced by exposure to the cholinergic receptor agonist carbachol in the hippocampus, which depends on NMDAR activation (Flores-Hernandez et al., 2009).

In addition, NMDAR is also regulated by ERK signaling pathway. In the spinal cord, CCL2 rapidly enhances NMDAR-induced neuronal electrical currents through the ERK-Glun2B pathway, thereby promoting pain sensitivity (Zhang H. et al., 2020). Related studies have found that CXCR7 can control the synaptic activity of hippocampal granular cells to regulate seizures. CXCR7 regulates GluN2A expression on the cell membrane by activating ERK1/2, thereby selectively modulating NMDAR-mediated synaptic neurotransmission in hippocampal granular cells (Xu T. et al., 2019). Therefore, CXCR7 may regulate seizures and become a new target for antiepileptic therapy by regulating the cell membrane expression of NMDAR. Some studies find that icaritin (ICT) has a neuroprotective effect on glutamate-induced neuronal damage and its mechanism may be associated with inactivating GluN2B-containing NMDAR by ERK/DAPK1 pathway (Liu et al., 2021). Meanwhile, DAPK1 interacts with NMDAR involved in glutamate-induced neurological events during sudden physiopathologic conditions in the brain (DeGregorio-Rocasolano et al., 2020). Inhibition of DAPK1 results in the phosphorylation and surface normalization of GluN2B expression outside the synapse (Schmidt et al., 2020).

Redox modulation of cysteine residues is one of the post-translational modifications of NMDAR. HCY accumulation in the human plasma, known as hyperhomocysteinemia, can exacerbate neurodegenerative diseases and act as a persistent NMDAR agonist (Ivanova M. et al., 2020). Meanwhile, HCY activates GluN2 subunit-dependent redox regulation of NMDAR by the reduction of NMDAR disulfide (Sibarov et al., 2020). The protein disulfide isomerase (PDI) binds to NMDAR in chronic epileptic rats and increases the mercaptan content on recombinant GluN1 protein (Kim et al., 2017). In fact, PDI plays a crucial role in catalyzing disulfide bond formation, reduction, and isomerization (Kim et al., 2017). Besides, H_2_S blocks the enhancement of neuronal excitability in the early hippocampal network by inhibiting voltage-gated sodium channels and NMDARs (Yakovlev et al., 2017). Thus, redox regulation of NMDAR may affect the occurrence and development of epilepsy and provide a new way for reducing the occurrence of epilepsy.

In epilepsy, some proteins and organisms can also affect NMDAR activity. As shown in the treatment of epilepsy, the inhibitory effect of β-hydroxybutyrate and acetone on NMDARs may underlie the therapeutic effects of the ketogenic diet in epilepsy (Pflanz et al., 2019). The interaction between the PCDH7 and the GluN1 subunit regulates the dendritic spine morphology and synaptic function, and it is associated with several CNS diseases including epilepsy (Wang Y. et al., 2020). In acute and chronic epileptic models, SPDI knockdown can inhibit seizure activity by nitrosylation-independent thiolation on NMDAR (Jeon and Kim, 2018). Inhibition of Nwd1 activity can reduce the hyperexcitability and phosphorylation of GluN2B in hippocampal neurons (Yang et al., 2019). Meanwhile, TMEM25 can also modulate the degradation of the GluN2B subunit and neuronal excitability (Zhang et al., 2019). Inhibition of acid-sensing ion channel 3 can regulate NMDAR function, thereby aggravating seizure severity (Cao et al., 2018). POSH could be a potential therapeutic target for epilepsy *via* increasing surface expression of NMDAR (Wang X. et al., 2017). Previous studies have shown that neuregulin1(NRG1)-ErbB4 signaling pathway may regulate the excitability of neurons and participate in primary epilepsy. NRG1-ErbB4 signaling can inhibit the phosphorylation of GluN2B, which has been detected in symptomatic human epileptic tissue (Zhu et al., 2017). In addition, the extracellular matrix protein SPARCL-1 also directly promotes synapse formation and NMDAR recruitment. In addition, SPARCL-1 might directly increase branches of dendrites, augment the numbers of synapse, and induce the formation of NMDARs, thereby increasing synaptic connectivity and reducing the risk for neurodegenerative disease (Chen et al., 2020b; Gan and Südhof, 2020). Leptin resists glutamate-induced excitotoxicity in HT22 hippocampal neurons and leptin also increases postsynaptic NMDAR currents to sensitize the nucleus of the solitary tract (NTS) neurons to vagal input (Jin et al., 2018; Neyens et al., 2020).

## Conclusion

In this review, we reviewed and elucidated the regulatory mechanisms of NMDAR and its role in the onset, development, and treatment of epilepsy. Increasing evidence suggests that NMDAR is closely related to epilepsy and the autoimmune encephalopathy. Synaptic NMDARs mainly mediate pro-survival and synaptic plasticity pathways, whereas extrasynaptic NMDARs are mostly responsible for glutamate-induced excitotoxicity. Meanwhile, different NMDAR subunit also has different physiological functions in epilepsy. Studying the role of various NMDAR subunits in epilepsy may be beneficial to understand epileptogenesis. At present, there are many ways to regulate NMDAR, but the regulatory mechanism of NMDAR in the onset and development of epilepsy is not fully understood. Therefore, targeting upstream and downstream signal pathways of NMDAR may be a new approach to inhibit seizures and slow the progression of epilepsy. This type of treatment is yet to be discovered and explored.

## Author Contributions

SC participated in experimental studies. SC and LF searched and sorted out the references and participated in drafting the manuscript. DX and ML coordinated and supervised the work, provided research direction, designed research plans, and modified the final drafts. All authors have carefully read and confirmed the final manuscript. All authors contributed to the article and approved the submitted version.

## Conflict of Interest

The authors declare that the research was conducted in the absence of any commercial or financial relationships that could be construed as a potential conflict of interest.

## Publisher’s Note

All claims expressed in this article are solely those of the authors and do not necessarily represent those of their affiliated organizations, or those of the publisher, the editors and the reviewers. Any product that may be evaluated in this article, or claim that may be made by its manufacturer, is not guaranteed or endorsed by the publisher.
